# RTP004 Peptide Binds to Botulinum Neurotoxin, Increases Cell Surface Binding, and Enhances Cellular SNAP-25 Cleavage

**DOI:** 10.3390/toxins18030134

**Published:** 2026-03-10

**Authors:** Andre F. Batista, Ratnesh Singh, Frank Lee, Shaoqiu Zhuo, Dmitri Leonoudakis, Conor J. Gallagher

**Affiliations:** Revance, 1222 Demonbreun St Suite 2000, Nashville, TN 37203, USA; andre.batista@revance.com (A.F.B.); ratnesh.singh@revance.com (R.S.); shaoqiu.zhuo@revance.com (S.Z.); dmitri.leonoudakis@revance.com (D.L.)

**Keywords:** botulinum neurotoxin type A, daxibotulinumtoxinA, neuromodulator, peptides, protein transduction domain, RTP004, SNAP-25

## Abstract

DaxibotulinumtoxinA for injection (DAXI) is a botulinum neurotoxin (BoNT) drug product comprising the 150 kDa pure BoNT/A1 as the drug substance formulated with a proprietary stabilizing excipient, RTP004. We hypothesized that RTP004 facilitates localization of BoNT/A1 to the neuronal membrane, resulting in increased BoNT internalization and cleavage of the synaptosomal-associated protein of 25 kDa (SNAP-25) within synaptic terminals. We characterized the interaction between RTP004 and BoNT/A1 using in silico and in vitro techniques. In vitro analyses revealed that negative charges on the BoNT/A1 surface were located on the light chain (LC, the catalytic domain) and the C-terminus of the heavy chain (H_C_, the receptor-binding domain), potentially providing sites for interaction with the positively charged RTP004 peptide. RTP004 bound to BoNT/A1, but not to human serum albumin (HSA), in both static and dynamic conditions. RTP004, not HSA, enhanced binding of BoNT to artificial membranes and RTP004 dissociated from BoNT under conditions that mimicked physiological conditions of the synaptic vesicle. RTP004 also increased binding of BoNT to the synaptosomal cell membrane and enhanced cleavage of SNAP-25 in a dose-dependent manner. These findings demonstrate that RTP004, not the excipient HSA common in other BoNT/A1 drug products, enhances binding of BoNT to the cell surface, facilitates internalization of BoNT into the cell, and increases SNAP-25 cleavage.

## 1. Introduction

Botulinum neurotoxins (BoNTs) are first-line symptomatic therapy for a number of debilitating medical conditions such as cervical dystonia, focal spasticity, blepharospasm, overactive bladder, hyperhidrosis, and chronic migraine, and are also used for a variety of aesthetic indications [1,2]. Produced primarily by the anaerobic bacteria *Clostridium botulinum* [3,4], BoNTs disrupt neuromuscular or neuroglandular transmission by the cleavage of presynaptic proteins, preventing exocytosis of acetylcholine, thus reducing muscular or secretory activity [5,6]. BoNTs are classified into seven established serotypes (A–G) based on differences in antigenicity, and within these serotypes into multiple subtypes based on their amino acid sequence variability. BoNT serotype A1 (BoNT/A1), which is the most potent serotype, has the longest duration of proteolytic activity [7], and has been the most extensively studied and most extensively developed BoNT for medical uses.

BoNT/A1 is produced as an inactive, single polypeptide chain of 150 kDa, which is nicked by an endoprotease to create a 100 kDa heavy chain (HC) and a 50 kDa light chain (LC) covalently linked by an inter-chain disulfide bond [6]. The HC contains two subdomains: the C-terminal region (H_C_), which mediates receptor binding, and the N-terminal region (H_N_), which mediates translocation of the LC across endosomal membranes. The LC contains a zinc-binding motif and functions as a metalloprotease that selectively and specifically cleaves synaptosomal associated protein of 25 kDa (SNAP-25), a component of the soluble N-ethylmaleimide-sensitive factor attachment protein receptor (SNARE) complex of proteins responsible for the docking and release of synaptic vesicles from the presynaptic nerve terminal [8,9].

The neuronal cell surface is coated by negatively charged polysialogangliosides, phosphatidylserines, phosphatidylinositols, and phosphatidylglycerols [10], including GT1_b_, which serve as low-affinity receptors for BoNT/A1 [11,12]. Based on surface charge distribution, the 150 kDa BoNT/A1 molecule is predicted to possess a dipole in which a more positively charged C-terminus facilitates orientation of the H_C_ to the negatively charged neuronal membrane [13]. Binding of a BoNT to low-affinity ganglioside receptors creates a reservoir of neurotoxin close to the neuronal surface. This reservoir increases the probability of the neurotoxin interacting with the luminal domain of the high-affinity BoNT/A1 receptor, synaptic vesicle glycoprotein 2 isoforms A–C (SV2A-C), which is exposed following membrane fusion and exocytosis of synaptic vesicles [12,14,15,16]. When bathed in BoNT/A1, an increased frequency of phrenic nerve stimulation leads to an increased rate of hemidiaphragm paralysis [17], suggesting that exposure to SV2 and SV recycling may be a rate-limiting step in the physiological activity of BoNT/A1.

Currently, six BoNT/A1 products have been approved by the US Food and Drug Administration and are commercially available [18]. The active pharmaceutical ingredients (BoNT/A1) in these products are stated by their manufacturers to range in molecular weight from 150 to 900 kDa based on the degree of purification of the core neurotoxin from the native neurotoxin-associated proteins. DaxibotulinumtoxinA for injection (DAXI; DAXXIFY^®^, Revance Therapeutics, Inc., Nashville, TN, USA) is a recently approved BoNT/A1 product consisting of the purified 150 kDa neurotoxin, the surfactant polysorbate 20, histidine buffers, and trehalose [19]. In addition, unlike other BoNT/A1 drug products that commonly use human serum albumin (HSA) as a stabilizing excipient, DAXI is formulated with a proprietary stabilizing excipient, RTP004. RTP004 is a synthetic 35-amino acid (~5 kDa) peptide consisting of a predominantly basic amino acid sequence (RKKRRQRRRGKKKKKKKKKKKKKKKGRKKRRQRRR) with two distinct domains: a core domain comprising 15 consecutive lysine residues, flanked at both ends by a 10-amino acid protein transduction/cell-penetrating peptide (CPP) domain [20]. RTP004 was designed as a lysine-rich, CPP-containing excipient intended to associate noncovalently with BoNT/A1 to enhance formulation stability and facilitate cellular uptake [20].

Clinically, the median duration of efficacy with DAXI has been demonstrated to be 24 weeks for treatment of glabellar lines and 20–24 weeks for treatment of cervical dystonia, which is longer than other products such as onabotulinumtoxinA (Botox^®^, AbbVie, Dublin, Ireland), which typically has a duration of 12–16 weeks [21]. When used clinically, BoNTs are dosed in units of biological activity, which are determined by the mouse median intraperitoneal lethal dose (LD_50_). However, because of the proprietary nature of the LD_50_ assay for each product/manufacturer and the absence of a World Health Organization international BoNT/A standard, the units of one product are not interchangeable with those of another [22]. As an example, the approved dose of DAXI for the aesthetic indication is 40 U, which contains ~0.18 ng of core neurotoxin, whereas the approved dose of onabotulinumtoxinA is 20 U, which has also been reported to contain ~0.18 ng of core neurotoxin [22,23]. A comparative phase 2 study for glabellar lines with 20 U of onabotulinumtoxinA and 20 U of DAXI showed that both BoNTs had a similar clinical profile in terms of efficacy and duration of effect, despite DAXI containing approximately half the amount of core neurotoxin reported for a 20 U onabotulinumtoxinA dose [24]. The fact that a similar clinical profile was observed with significantly less neurotoxin suggests that the DAXI formulation has an important role in the clinical performance of the product, perhaps resulting in a more efficient use of neurotoxin.

In this series of studies, we aimed to characterize the interaction between RTP004 and BoNT/A1 using both in silico and in vitro techniques. Our hypothesis was that the RTP004 peptide functions not only as a stabilizing excipient but may also interact with BoNT/A1 and facilitate its localization to the neuronal membrane, resulting in increased BoNT internalization and cleavage of SNAP-25 within neurons.

## 2. Results

### 2.1. Electrostatic Potential Analysis of BoNT/A1

**Figure 1:**
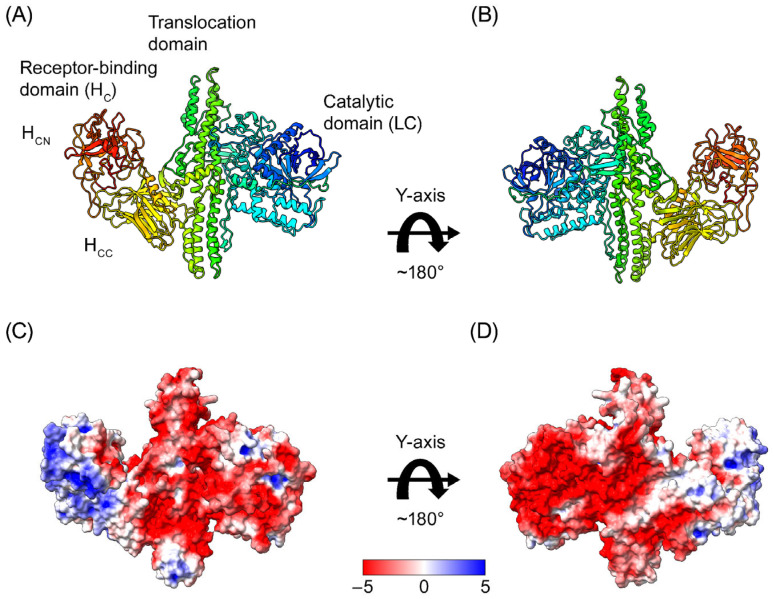
Structure and charge distribution on the surface of the BoNT/A1 holotoxin. (**A**) Crystal structure and domain composition of BoNT/A1 (PDB 3BTA, Lacy et al. [25]). (**B**) Rotational view of BoNT/A1, rotated ~180° along the *y*-axis relative to (**A**). (**C**) Charge distribution on the BoNT/A1 surface at pH 7.4. (**D**) Rotational view of BoNT/A1, rotated ~180° along the *y*-axis relative to (**C**). Negatively charged residues are colored red, positively charged residues are colored blue, and neutral residues are colored white. Abbreviations: H_C_—heavy chain receptor-binding domain; H_CC_—C-terminal part of H_C_; H_CN_—N-terminal part of H_C_; LC—light chain.

The crystal structure of the BoNT/A1 holotoxin was previously determined (PDB code: 3BTA) [25], revealing distinct functional domains, including the catalytic LC, the translocation domain (H_N_), and the receptor-binding domain (H_C_) (Figure 1A,B). The electrostatic surface map visualized using Chimera X demonstrates an uneven distribution of negatively (red) and positively (blue) charged areas on the BoNT/A1 surface (Figure 1C,D). Negatively charged residues were present across the BoNT/A1 surface with higher concentrations on the LC (the catalytic domain) and translocation domain than on the C-terminal portion of the receptor-binding domain, which was mostly positively charged or neutral. This reflects an intrinsic dipole first described by Fogolari et al. [13].

**Figure 2:**
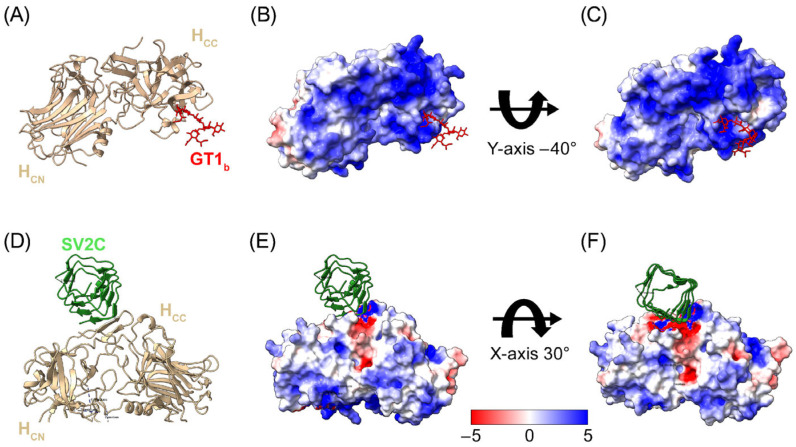
Structure and charge distribution on the surface of the BoNT/A1 receptor domain in complex with its receptors GT1_b_ and SV2C. (**A**) Crystal structure of the BoNT/A1 receptor domain with GT1 (PDB code: 2VU9). (**B**) Charge distribution of the BoNT/A1 surface with GT1_b_ binding at pH 7.4. (**C**) Rotational view of the BoNT/A1 surface, rotated −40° along the *y*-axis relative to (**B**). (**D**) Crystal structure of the BoNT/A1 receptor domain with SV2C (PDB code: 4JRA). (**E**) Charge distribution of the BoNT/A1 surface with SV2C binding at pH 7.4. (**F**) Rotational view of the BoNT/A1 surface, rotated ~30° along the *x*-axis relative to E. Negatively charged residues are colored red, positively charged residues are colored blue, and neutral residues are colored white. Abbreviations: GT1_b_—sialylated glycosphingolipid; H_CC_—C-terminal part of H_C_; H_CN_—N-terminal part of H_C_; SV2C—synaptic vesicle glycoprotein 2C.

To determine whether potential binding of the RTP004 peptide to the C-terminal region of the BoNT/A1 HC receptor-binding domain (H_C_) could hinder the interaction between the neurotoxin and its receptors, we examined the charge distribution of the C-terminal region of the BoNT/A1 HC while in complex with the GT1_b_ and SV2 receptor isoform C (SV2C) binding sites (Figure 2) [12,26,27].

The electrostatic map revealed that the low-affinity GT1_b_ binding site and the high-affinity SV2C binding site were generally restricted to the positively charged (blue) areas on the BoNT/A1 surface, suggesting that the highly positively charged RTP004 may not hinder SV2C receptor binding.

### 2.2. Binding of BoNT/A1 to RTP004 or HSA

**Figure 3:**
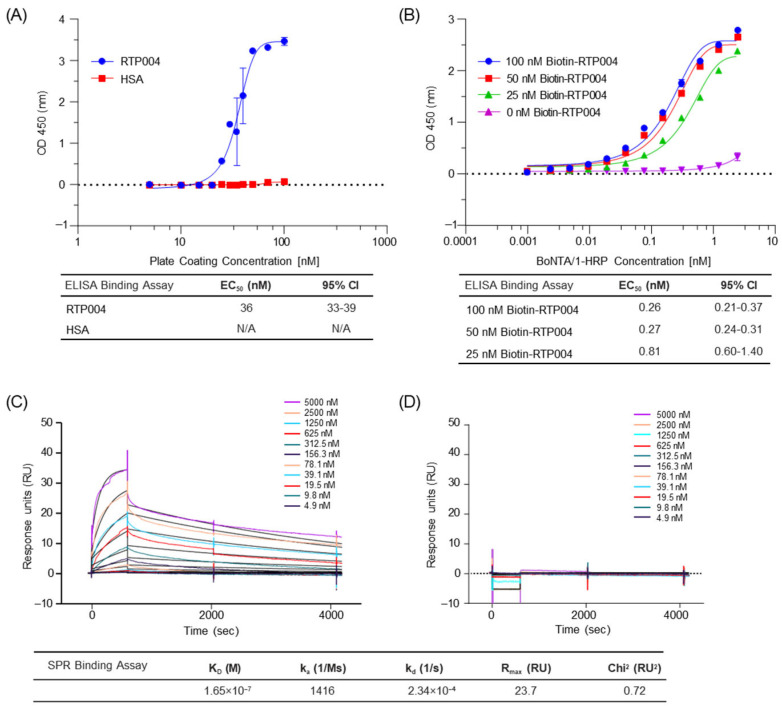
BoNT/A1 binding to RTP004 in static and dynamic binding environments. (**A**) ELISA-based binding assay showing HRP-labeled BoNT/A1 (3.3 nM) binding to RTP004 or HSA-coated plates. Data are the mean of three independent experiments ± standard deviation. (**B**) ELISA-based binding assay comparing increasing concentrations of HRP-labeled BoNT/A1 binding to three different concentrations of RTP004 (25 nM, 50 nM or 100 nM). Data are the mean of representative duplicates ± standard deviation. (**C**) SPR-Sensorgram for the determination of binding kinetics of BoNT/A1 binding to the RTP004-coated sensor chip. (**D**) SPR-Sensorgram for the determination of binding kinetics of BoNT/A1 binding to the HSA-coated sensor chip. Abbreviations: BoNT/A1—botulinum neurotoxin type A; CI—confidence interval; EC_50_—half-maximal effective concentration; ELISA—enzyme-linked immunosorbent assay; HRP—horseradish peroxidase; HSA—human serum albumin; N/A—not applicable; OD—optical density; RU—response units; SPR—surface plasmon resonance.

Enzyme-linked immunosorbent assay (ELISA) and surface plasmon resonance (SPR) were used to evaluate the interactions between BoNT/A1 and RTP004 or HSA. ELISA demonstrated binding of HRP-BoNT/A1 (3.3 nM) to increasing concentrations of immobilized RTP004 with an EC_50_ of 36 nM, whereas no detectable interaction was observed with immobilized HSA (Figure 3A). In this assay, RTP004 or HSA was immobilized on the plate surface at increasing coating concentrations, and a fixed concentration of HRP-labeled BoNT/A1 (3.3 nM) was added to each well to assess binding. Because ELISA relies on adsorption of RTP004 to the surface of the assay plate, the measured response may be influenced by surface density of immobilized peptide. To assess the impact of coating concentration on the observed signal and calculated EC_50_ values, assay plates were coated with Biotin-RTP004 at higher concentrations (25, 50, and 100 nM). For each coating condition, increasing concentrations of HRP-labeled BoNT/A1 were added to the plate, to evaluate binding (Figure 3B). Under these conditions, EC_50_ values of 0.26, 0.27 and 0.81 nM were obtained for HRP-labeled BoNT/A1 at Biotin-RTP004 coating concentrations of 100, 50 and 25 nM, respectively (Figure 3B). Increasing coating concentration was associated with increased signal magnitude and lower apparent EC_50_ values, thus demonstrating an avidity effect.

SPR was conducted to evaluate the interaction between BoNT/A1 and RTP004. Injection of increasing concentrations of BoNT/A1 over an RTP004-coated CM5 sensor chip resulted in concentration-dependent binding with an apparent K_D_ of 165 nM (Figure 3C). To assess specificity, BoNT/A1 was injected over an HSA-immobilized surface under the same assay orientation and concentration range. No detectable binding was observed (Figure 3D).

RTP004 was immobilized to 187 response units (RU) using 1-ethyl-3-(3-dimethylaminopropyl)carbodiimide (EDC)/N-hydroxysuccinimide (NHS)-mediated amine coupling to the carboxymethylated dextran matrix of the CM5 chip. This coupling chemistry can result in random covalent binding through accessible primary amines, which can limit the accessibility of immobilized RTP004 peptide. Consistent with this, the observed Rmax (23.7 RU) was substantially lower than the theoretical Rmax (~5600 RU). To further evaluate the impact of ligand density on binding behavior, additional SPR experiments were conducted using varying levels of immobilized RTP004 and titration of HRP-labeled BoNT/A1 as the analyte. It was found that increasing immobilized RTP004 density resulted in a lower calculated K_D_ value, consistent with surface density-dependent avidity effects rather than changes in molecular affinity (Figure S1).

### 2.3. Effect of RTP004 on Binding of BoNT/A1 to Artificial Membranes

**Figure 4:**
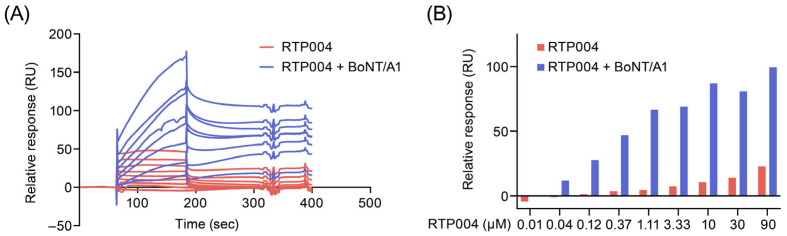
Effect of RTP004 on BoNT/A1 binding to artificial membranes. (**A**) SPR-Sensorgram of RTP004 and preincubated RTP004 + BoNT/A1 binding to biotinylated polymerized liposomes captured on the sensor chip. The concentration of RTP004 was titrated from 90 to 0 µM and the cycles with BoNT/A1 had a concentration of 5 µM. (**B**) Graphical representation of the binding levels in response units of RTP004 alone (red) and preincubated RTP004 + BoNT/A1 (blue). Abbreviations: BoNT/A1—botulinum neurotoxin type A; RU—response units.

To establish a more physiologically relevant condition, we used an artificial lipid bilayer for SPR analysis to assess the effect of RTP004 on BoNT/A1 binding. A C1 sensor chip was prepared by immobilizing streptavidin onto two adjacent flow cells via standard amine coupling. The primary flow cell served as a reference surface, consisting of immobilized streptavidin alone, while the secondary flow cell was utilized for the site-specific capture of biotinylated liposomes (polymerized liposomes). These biotinylated liposomes (PolyPIPosomes; Echelon Biosciences) are polymerized liposome-like nanoparticles (~200 nm) composed of a defined phosphoinositide lipid mixture containing 5% PI(3)P and supplied as a 1 mM total lipid formulation, with an incorporated biotin tag to enable streptavidin-mediated surface capture. To account for non-specific binding and bulk refractive index shifts, the experimental data were processed by subtracting the reference response from the active surface signal. BoNT/A1 preincubated with 0 to 90 µM RTP004 (0 to 450 µg/mL) was injected as the analyte. BoNT/A1 preincubated with RTP004 demonstrated a dose-dependent increase in binding to the captured polymerized liposomes (Figure 4A,B, blue). To ensure that the binding response was not due to unbound RTP004, the latter was also injected as analyte at the same range of concentrations to characterize its binding to the captured polymerized liposomes. Binding of RTP004 to the polymerized liposomes was observed, but at a substantially lower level compared to the binding observed with BoNT/A1 preincubated with RTP004. Consequently, the observed binding signal is confirmed to originate from the BoNT/A1-RTP004 complex, not RTP004 alone (Figure 4A,B, red).

### 2.4. Dissociation of RTP004 from BoNT/A1

To analyze the dissociation of RTP004 from BoNT/A1, SPR analyses were conducted under conditions that mimicked the pH (5.5 to 4.5) and osmolarity (NaCl concentrations from 0 to 175 mM) of the endosome. BoNT/A1 was directly immobilized onto C1 sensor chips and RTP004 was injected as analyte. BoNT/A1 was immobilized to a response signal of 348 RU, and an EDC/NHS blank immobilization was used as a reference. To account for non-specific binding and bulk refractive index shifts, the final binding data were reported as a subtracted sensorgram. The average maximal bound RU after peptide was injected and washed for every cycle was 31.0 RU with a standard deviation of 0.9 RU. Representative sensorgram illustrating these dissociate profiles are provided in Figure S2.

**Figure 5:**
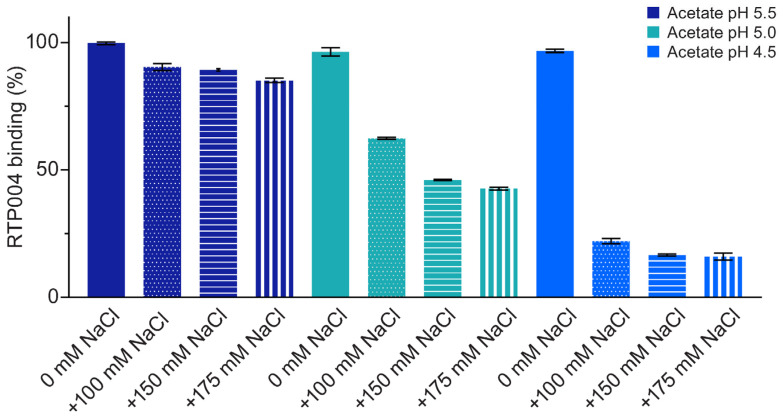
Effect of pH and salt conditions on the dissociation of RTP004 from BoNT/A1. Bar graph of the percentage of RTP004 bound to BoNT/A1 after washing conditions, normalized to the RTP004 bound for each cycle. BoNT/A1 was directly immobilized to C1 sensor chips and RTP004 was injected as analyte at a concentration of 2 µM for each cycle. Data represent the mean ± standard deviation of five independent experiments. Abbreviations: NaCl—sodium chloride.

RTP004 dissociated from BoNT/A1 at a lower pH and with increasing sodium chloride (NaCl) concentration (Figure 5). RTP004 did not dissociate from BoNT/A1 under any of the pH conditions tested in the absence of NaCl. At pH 5.5 with NaCl, there was very little evidence of dissociation, with the percentage of RTP004 binding to BoNT/A1 ranging from 91.3% to 84.1% across all NaCl concentrations. At pH 5.0, the percentage of RTP004 binding to BoNT/A1 decreased from 62.9% at 100 mM NaCl to 42.1% at 175 mM NaCl, indicating some dissociation of RTP004 from BoNT/A1. At pH 4.5 dissociation was more evident, with <50% of RTP004 binding BoNT/A1at all NaCl concentrations.

### 2.5. Effect of RTP004 on Binding of BoNT/A1 Hc to Rat Synaptosomal Cell Membrane

**Figure 6:**
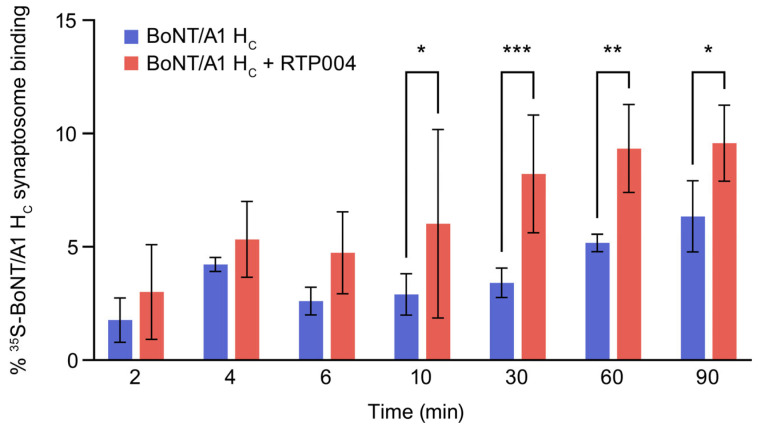
Binding of BoNT/A1 receptor domain H_C_ to synaptosomes. The binding of ^35^S-labeled BoNT/A1 H_C_ to rat brain-derived synaptosomes was quantified in the presence or absence of RTP004. Data represent the mean ± standard deviation of six independent experiments. * *p* < 0.05, ** *p* < 0.01, *** *p* < 0.001; two-way analysis of variance followed by Šídák’s multiple comparisons test. Abbreviations: BoNT/A1—botulinum neurotoxin type A; H_C_—receptor-binding domain.

Using synaptosome preparations from rat brain, we tested whether the presence of RTP004 increased the amount of BoNT/A1 receptor-binding domain (H_C_) that could bind to the synaptosomal surface. The amount of ^35^S-labeled BoNT/A1 H_C_ that bound to the synaptosomes was significantly enhanced in the presence of RTP004 compared with BoNT/A1 H_C_ alone at 10, 30, 60, and 90 min (Figure 6).

### 2.6. Effect of RTP004 on Binding of BoNT/A1 to the Surface of Human Neuroblastoma Cells and on SNAP-25 Cleavage

**Figure 7:**
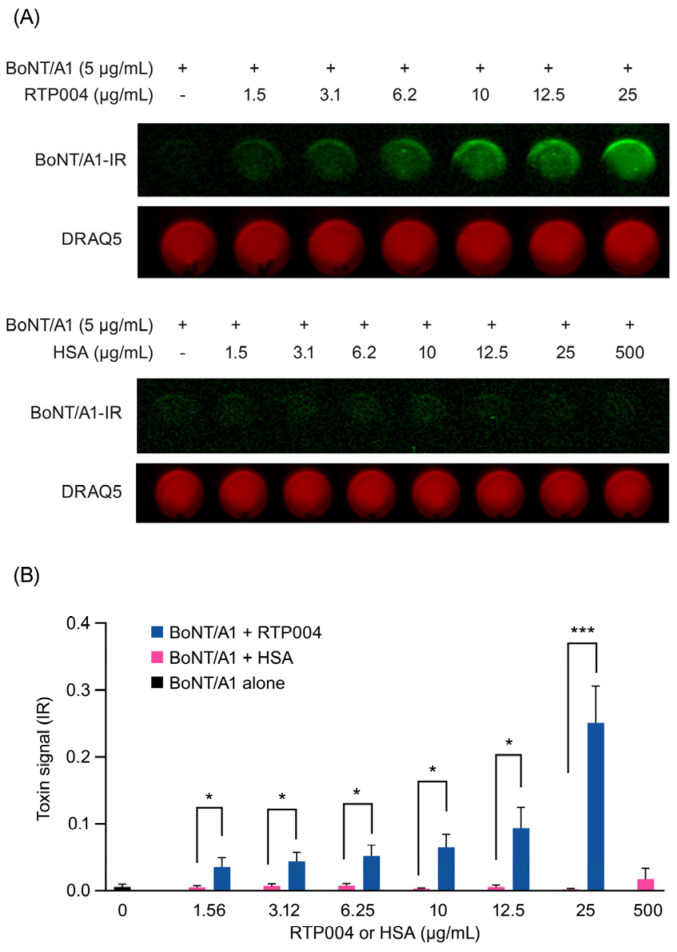
Effect of RTP004 on binding of BoNT/A1 to human neuroblastoma SiMa cell surfaces. (**A**). Representative images from IR790-labeled BoNT/A1 (green) and DRAQ5 nuclear staining (red, used as a control for cell confluency) staining in the presence of RTP004 and HSA. (**B**). Quantification of IR790-labeled BoNT/A1 binding to SiMa cells normalized to the DRAQ5 signal from the same well. Data represents the mean ± standard error of the mean of 3 independent experiments. * *p* < 0.05, *** *p* < 0.001; Student’s *t*-test. Abbreviations: BoNT/A1—botulinum neurotoxin type A; HSA—human serum albumin; IR—infrared.

To examine whether RTP004 could enhance binding of BoNT/A1 to neuroblastoma cells, undifferentiated SiMa cells were co-treated with a fixed amount of infrared dye-labeled BoNT/A1 (5 µg/mL = 33.3 nM) and increasing amounts of RTP004 or HSA, respectively (Figure 7). The results demonstrated that BoNT/A1 binding to the cell surface significantly increased with increasing concentrations of RTP004, but not with HSA.

**Figure 8:**
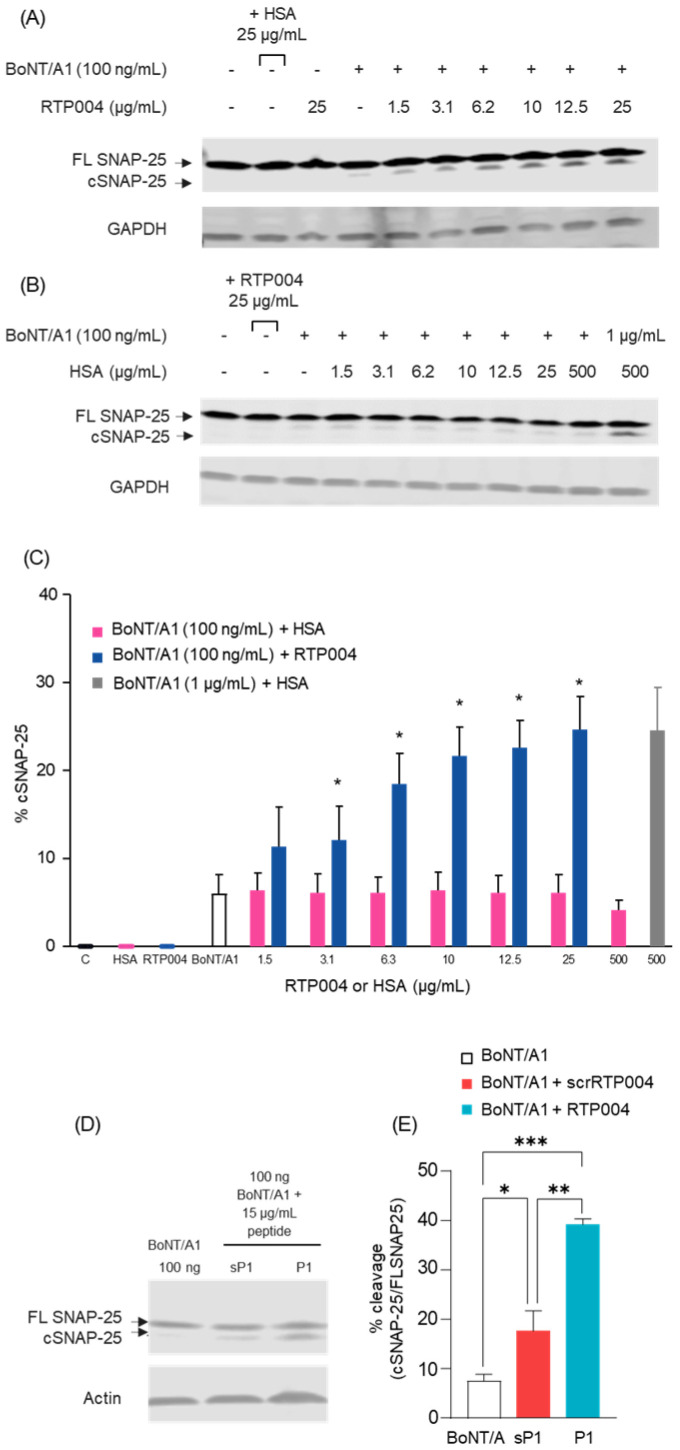
RTP004 enhances BoNT/A-mediated SNAP-25 cleavage in a sequence-dependent manner. (**A**) SiMa cells were co-treated with BoNT/A1 (100 ng/mL or 0.67 nM) alone, or BoNT/A1 + RTP004, or (**B**) BoNT/A1 + HSA. Cleavage of SNAP-25 was detected by immunoblot using antibodies that recognize both intact and cleaved SNAP-25 protein. GAPDH served as the internal control for loading. (**C**) Compiled immunoblot densitometry quantification of the percentage increase in cleaved SNAP-25. Data represent the mean ± standard error of the mean of seven experiments. * *p* < 0.05; Student’s *t*-test. (**D**) Representative immunoblot of SNAP-25 cleavage in SiMa cells treated with BoNT/A1 alone, in the presence of RTP004 (P1) or a charge-matched scrambled peptide (scrRTP004; sP1) at 15 µg/mL. Cleaved and full-length SNAP-25 bands were detected as in panel A and B, with actin as a loading control. (**E**) Compiled immunoblot densitometry quantification of the percentage increase in cleaved SNAP-25. While scrRTP004 produced a modest increase in cSNAP-25 (132% increase compared to BoNT/A1 alone), RTP004 induced a significantly greater effect (417% increase compared to BoNT/A1 alone). Data represent mean ± SEM from three independent experiments. Statistical significance was determined by one-way ANOVA followed by Holm–Šídák multiple comparisons test, with all pairwise comparisons performed. * *p* < 0.05; ** *p* < 0.01; *** *p* < 0.001. Abbreviations: BoNT/A1—botulinum neurotoxin type A; C—vehicle control; cSNAP25—cleaved/cleavage product of SNAP-25; FL SNAP-25—full-length (uncleaved) SNAP-25; GAPDH—glyceraldehyde 3-phosphate dehydrogenase; HSA—human serum albumin; scrRTP004 (sP1)—scrambled RTP004 peptide; SNAP-25—synaptosomal associated protein of 25 kDa.

A SNAP-25 cleavage assay in SiMa cells was used to determine the effect of RTP004 on BoNT/A1 function after binding to the cell surface. Increasing concentrations of RTP004 were associated with an increase in cleaved SNAP-25 compared with SiMa cells treated with BoNT/A1 alone (Figure 8A,C). In contrast, there was no increase in cleaved SNAP-25 with increasing amounts of HSA in the presence of a fixed amount of BoNT/A1 (Figure 8B,C).

To decouple CPP-facilitated effects from non-specific charge effects we compared RTP004 with a scrambled RTP004 peptide (scrRTP004; KRKKRGKKRKKKRQKRRKKRKKKRKKKGRKRRQRK) that retains equal cationic charge but lacks an organized protein transduction domain. SiMa cells were treated with BoNT/A1 alone or in combination with RTP004 or scrRTP004 (15 µg/mL), and SNAP-25 cleavage was assessed by immunoblot (Figure 8D). Quantitative densitometry demonstrated that the ratio of cleaved SNAP-25 normalized to total SNAP-25 was significantly greater with RTP004 compared with BoNT/A1 plus sRTP004 and BoNT/A1 alone (Figure 8E, *p* < 0.01). Because both peptides are equally charged, these findings demonstrate that net positive charge alone is insufficient to account for the observed functional increase in cSNAP-25 and further demonstrates the requirement for the intact CPP sequence of RTP004.

## 3. Discussion

In this study, we characterized the interaction between RTP004 and BoNT/A1 using both in silico and in vitro techniques and further explored the potential for RTP004 to enhance neuronal cell binding of BoNT/A1 and facilitate its pharmacological activity. Our results demonstrated that RTP004 binds to BoNT/A1 under both static and dynamic binding conditions. RTP004, but not the common excipient HSA used in other BoNT/A1 formulations, enhanced binding of BoNT/A1 to the cell surface and increased the cleavage of SNAP-25 in a dose-dependent manner.

RTP004 is highly positively charged at physiological pH with a pI of 13.1, allowing it to form electrostatic interactions with negatively charged regions of biologically active molecules as well as extracellular structures such as neuronal surfaces and extracellular matrix proteins [19]. ELISA and SPR analyses revealed that there is an avidity-driven interaction between RTP004 and BoNT/A1. Such avidity effects were much stronger when RTP004 was immobilized on avidin rather than randomly coated on the surface of the plastic plate. It is possible that uniformly captured biotin-RTP004 behaves as if in solution resulting in a higher effective surface concentration with better availability to bind to the toxin in contrast to the peptide adsorbed onto the plate surface. This could account for the difference in EC_50_ obtained in different assays (Figure 3).

The RTP004 peptide serves an excipient function (performed by HSA in other formulations) by preventing self-aggregation of BoNT/A1 molecules and enhancing thermal stability of the BoNT/A1 [28]. Because HSA is a much larger protein than RTP004 with a pI of 4.7, it was not expected to form a strong charge-based interaction with BoNT/A1. This was confirmed by both SPR and ELISA in our experiments.

Analysis of charge distribution across the BoNT/A1 surface revealed multiple potential sites for interaction with RTP004. Negative charges were unevenly distributed across the BoNT/A1 surface, with more residues on the LC and fewer residues on the HC. However, as the HC contains the receptor-binding domain, it was conceivable that binding of RTP004 to the HC may occlude either the ganglioside or SV2 binding sites. Our in silico analyses showed that the low-affinity GT1_b_ binding site and the high-affinity SV2C binding site on the receptor-binding domain contained predominantly positively charged or neutral residues, suggesting that RTP004 is unlikely to sterically hinder receptor binding.

After injection into a target muscle, BoNT/A1 typically binds with low affinity to GT1_b_ receptors present on the neuronal surface. This interaction maintains a reservoir of BoNT/A1 at the neuronal surface, where it is then available for binding to the SV2 receptor exposed during synaptic vesicle fusion [11,12,13,14,15]. The greater the reservoir of membrane-associated BoNT/A1, the more will be available for SV2 binding and subsequent internalization. Unbound or weakly bound toxins will be flushed from the area through interstitial fluid convection, lowering the local concentration of toxins at the neuronal membrane [29]. It has been inferred indirectly that the half-life of toxins in the interstitium may range from minutes to a few hours [29].

Given the strong association between the RTP004 peptide and BoNT/A1, we hypothesized that the toxin–peptide complex may demonstrate a greater interaction with neuronal membranes than BoNT/A1 alone. We observed that this was the case in three systems of progressive complexity. Firstly, by using SPR we demonstrated that for a fixed concentration of BoNT/A1, there was an increase in toxin binding to polymerized liposomes with increasing concentrations of RTP004. Although polymerized liposomes can mimic the charge and curvature of real membranes, they lack the native constituents (proteins, glycoproteins, and glycolipids) that make up the cellular membrane [30]. Therefore, this result was verified in rat brain-derived synaptosomes, which contain isolated nerve terminals and are commonly used as a model for evaluating synaptic function [31]. Finally, we explored RTP004-facilitated binding of BoNT/A1 in a human neuroblastoma (SiMa) cell line. Again, we observed an increase in binding of a fixed concentration of IR790-labeled BoNT/A1 to the surface of SiMa cells with increasing concentrations of RTP004. Taken together, these data support a conclusion that BoNT/A1 binding to cell membranes is enhanced when associated with the RTP004 peptide. This may be attributable to the peptide conferring an increased positive charge to the toxin–peptide complex, facilitating binding to the negatively charged neuronal membrane. No increase in binding was observed in the presence of HSA, which does not bind to BoNT/A1. Based on these data, it is possible that the increased binding of the toxin–peptide complex at the membrane increases the reservoir of BoNT/A1 at the membrane and/or the length of time the BoNT/A1 remains associated with the membrane. As the uptake of BoNT/A1 is activity-dependent [32], a persistence of a pool of BoNT/A1 docked at the neuronal membrane would allow for a greater probability of encountering SV2 and internalization. Our finding that SNAP-25 cleavage increased in proportion to the amount of peptide is supportive of this hypothesis.

The inclusion of a charge-matched scrRTP004 control provides important mechanistic insight into the faciliatory effects of RTP004. While the cationic nature of these peptides contributes to interactions with the neuronal surface, the significant difference in SNAP-25 cleavage observed with intact RTP004 versus scrRTP004 demonstrates that sequence-specific CPP organization rather than positive charge alone is required to produce efficient cytosolic delivery of the catalytic domain of the toxin.

The contrast between the RTP004-containing BoNT/A1 formulation and HSA-containing formulations may be further illustrated by a clinical study that compared DAXI to onabotulinumtoxinA for the aesthetic treatment of glabellar frown lines [24]. In this study, a 20 U dose of onabotulinumtoxinA (reportedly containing ~0.18 ng core neurotoxin, plus albumin) was compared with 20 U of DAXI (which contains 0.09 ng core neurotoxin and the RTP004 peptide). At these dose levels, both products demonstrated a broadly similar clinical response, despite DAXI containing approximately half the core neurotoxin in the administered dose [33]. This implies a more efficient use of the administered neurotoxin, likely as a function of the formulation excipients, including the RTP004 peptide.

Several in vitro studies evaluating BoNT/A1 cleavage of SNAP-25 have applied BoNT/A1 at lower concentrations but for an extended period of time (up to 48 h) [34,35]. Prior to the initiation of these experiments, we optimized the concentration and duration of BoNT/A1 exposure to establish a minimum detectable SNAP-25 cleavage. Specifically, cells were treated with BoNT/A1 (100 ng/mL or 1 µg/mL) in the presence of increasing concentrations of RTP004 or HSA for a 1 h time period, followed by a washout and a 24 h incubation before measuring SNAP-25. As prolonged bath exposure of BoNT/A1 could mask any potential effect of the RTP004 peptide in increasing the affinity to the neuronal cells, and thus the downstream impact on SNAP-25 cleavage, we elected to minimize the exposure time. We followed exposure by a wash step to more closely mimic the in vivo condition where unbound toxin is washed from the injected area.

The failure of covalently linked CPP-based therapeutics in clinical trials has been attributed in large part to the low degree of endosomal escape those molecules can achieve. In most cases, <1% of the internalized protein can access the cytosol [36]. BoNT/A1 is exceptional in this regard, as it has an intrinsic endosomal escape mechanism. Given the high degree of affinity of the peptide for BoNT/A1, it is possible that the peptide remains associated with the toxin and limits the translocation of the LC from the endosome into the cytosol. This seems highly unlikely, as we have convincingly demonstrated that with increasing amounts of peptide, we see increased SNAP-25 cleavage in vitro and a robust response in patients receiving the product in clinical trials. Nonetheless, we sought to establish the conditions under which the peptide would dissociate from the toxin. Using SPR, we observed that the peptide dissociates from the 150 kDa BoNT/A1 under conditions that mimic physiological saline conditions and pH. At the lowest pH we examined, the peptide was >75% dissociated from the toxin at NaCl concentrations >100 mM. These conditions simulate the acidified endosomal environment and demonstrate that RTP004 likely dissociates from BoNT/A1 within the endosome and does not interfere with its enzymatic function.

We have shown that RTP004 enhances toxin binding at the neuronal cell surface, likely by boosting the number of electrostatic interactions between the toxin and the negatively charged gangliosides on the cell surface. The extracellular matrix of the muscle is composed of ground substance, with structure provided by polypeptides such as collagen and elastin, as well as glycosaminoglycans. These extracellular matrix proteins and glycosaminoglycans are replete with negatively charged phosphate groups and may bind the toxin–peptide complex, helping retain it within the injected muscle. This hypothesis is supported by the clinical observation that patients treated with DAXI experience a lower incidence of adverse events associated with neurotoxin diffusion to non-target tissues, compared with BoNT/A1 products formulated with conventional excipients such as albumin [19,24,37].

## 4. Conclusions

Here, we demonstrate that the cationic peptide RTP004 has a strong yet reversible association with BoNT/A1, likely due to electrostatic interaction. RTP004 facilitates binding of BoNT/A1 to neuronal membranes and dissociates from BoNT/A1 at acidic pH similar to the endosomal environment. Further, RTP004, but not a charge-matched scrambled peptide, increases BoNT/A1-induced SNAP-25 cleavage in neuronal cells. These data support a sequence-dependent mechanism in which the excipient RTP004, unlike HSA used in other BoNT/A1 formulations, facilitates neuronal synaptic membrane binding and internalization, and enhances functional activity of BoNT/A1, culminating in increased SNAP-25 cleavage.

## 5. Materials and Methods

### 5.1. In Silico Protein Surface Analysis and Image Generation

Crystal structures of BoNT/A1 (PDB code: 3BTA), H_C_A–GT1_b_ (PDB code: 2VU9), and H_C_A–SV2C (PDB code: 4JRA) were obtained from the Research Collaboratory for Structural Bioinformatics (RCSB) Protein Data Bank (https://www.rcsb.org, accessed on 15 December 2025). Each structure was processed using the PDB2PQR tool available on the Adaptive Poisson–Boltzmann Solver (APBS) webserver (v3.2.2; https://server.poissonboltzmann.org). Protonation states were assigned using PROPKA at pH 7.4, and atomic charges were calculated with the PARSE force field. The resulting PQR files were then used as input for the APBS tool to compute electrostatic potential maps. Electrostatic potentials were visualized using ChimeraX (v1.10.1) by mapping the APBS-generated potentials onto the molecular surfaces of the respective structures [38].

### 5.2. ELISA Binding

For all ELISA-based assays, an M5 or i3x plate reader (Molecular Devices, San Jose, CA, USA) was used to read absorbance at an optical density of 450 nm, indicating the amount of bound BoNT/A1. HRP was conjugated to recombinantly expressed 150 kDa BoNT/A1 (Toxogen, Hannover, Germany) using the Mix-n-Stain^TM^ HRP Antibody Labeling Kit (Biotium, Fremont, CA, USA) according to the manufacturer’s protocol. For the binding assay in Figure 3A, a 96-well MaxiSorp flat-bottom plate was coated with 100 μL per well of increasing concentrations of RTP004 (Revance Therapeutics, Nashville, TN, USA) or HSA (Fujifilm, Tokyo, Japan) from 0 to 500 nM in phosphate-buffered saline (PBS) and incubated for 1 h at 37 °C. For the binding assay in Figure 3B, a 96-well MaxiSorp flat-bottom plate was coated with 100 μL per well of 10 μg/mL of avidin (Thermo Fisher Scientific) for 1 h at 37 °C. After incubation, the plate was washed four times with 350 μL per well of PBS with 0.05% Tween 20 (PBST) and then blocked with 300 μL/well of ELISA blocking buffer (Thermo Fisher Scientific, Waltham, MA, USA) for 1 h at 37 °C. After blocking, the plate was washed four times in 350 μL/well PBST followed by incubation with 0, 25, 50, or 100 nM of Biotin-RTP004 (AnaSpec, Fremont, CA, USA) in PBS for 30 min at ambience. HRP-conjugated neurotoxin (100 μL/well at 3.3 nM for Figure 3A and varying concentrations from 0 to 2.5 nM for Figure 3B) in blocking buffer was added. The plate was incubated for 1 h at either 37 °C or ambient temperature, and washed another four times in PBST followed by the addition of 100 μL/well of 3,3′,5,5′-Tetramethylbenzidine (TMB) Liquid Substrate System for ELISA (Thermo Fisher Scientific). The reaction was continued for 5–15 min depending on assays to allow color to develop, was stopped by the addition of 50 μL/well of a 1 M sulfuric acid solution, and the absorbance was read at 450 nm. Data were analyzed using GraphPad Prism (version 10.1.2) and EC_50_ was obtained by four or five parameters least square fit.

### 5.3. SPR Direct Binding Assays

For all SPR-based binding assays, the Biacore system T200 (Cytiva, Marlborough, MA, USA) was used. The direct binding interaction analysis of BoNT/A1 to RTP004 was carried out on a CM5 Series S sensor chip (Cytiva, cat# 29149603). The CM5 chip was coated with RTP004 (2 to 0.5 µg/mL in 10 mM acetate buffer, pH 5.0) by direct immobilization using N-(3-dimethylaminopropyl)-N’-ethylcarbodiimide (EDC) and N-hydroxysuccinimide (NHS), applying amine coupling chemistry. In brief, a 1:1 mixture of NHS and EDC was injected, followed by injection of RTP004 and blocking of residual binding sites with 1 M ethanolamine. A blank immobilization, where the EDC and NHS amine coupling chemistry was applied without the RTP004 peptide, was used as the reference flow cell. The final response of RTP004 bound ranged from 50 to 200 response units (RU). To establish binding kinetics, eleven 2-fold dilutions of BoNT/A1 (Revance Therapeutics) were performed (4.9 to 5000 nM in HBS-EP+ [10 mM HEPES, 150 mM NaCl, 3 mM EDTA, and 0.05% Surfactant P20, pH 7.4, Cytiva cat# BR100669]). The binding analysis was carried out at 25 °C with HBS-EP+ as running buffer. Before the run, the chip was preconditioned with 200 mM NaOH and 0.5% sodium dodecyl sulfate (SDS), and three blank startup injections were performed. BoNT/A1 at each concentration was injected at 30 µL/min over the immobilized RTP004 for a contact time of 600 s and dissociation time of 3600 s. 4 M MgCl_2_ was used to regenerate the chip between injections and was injected at 30 µL/min for 30 s followed by a buffer wash and a 300 s stabilization period.

The direct binding interaction analysis of BoNT/A1 to HSA (Fujifilm, Tokyo, Japan) was carried out on a CM5 Series S sensor chip. The CM5 chip was coated with HSA (10 µg/mL in 10 mM acetate buffer, pH 5.0) by direct immobilization using the same method as described above. A blank immobilization, where the EDC and NHS amine coupling chemistry was applied without HSA, was used as the reference flow cell. The final response of bound HSA was 2528 RU. To establish binding kinetics, eleven 3-fold dilutions of BoNT/A were performed (4.9 to 5000 nM in HBS-EP+ [10 mM HEPES, 150 mM NaCl, 3 mM EDTA, and 0.05% Surfactant, Polysorbate 20 (PS20), pH 7.4, Cytiva cat# BR100669]). Binding kinetics were analyzed as described above.

### 5.4. SPR Binding Assay to Polymerized Liposomes

Streptavidin (20 µg/mL Millipore Sigma, Burlington, MA, USA, cat# 18-973-01MG) was directly immobilized onto Series S sensor chip C1 (Cytiva, cat# BR100535) using the same amine coupling as described above. Streptavidin was immobilized onto flow cells 1 and 2 of a C1 sensor chip to facilitate surface functionalization. Flow cell 1 (FC1) was maintained as a reference surface, whereas flow cell 2 (FC2) was functionalized with biotinylated polymerized liposomes. This configuration allowed for the subtraction of non-specific binding effects during subsequent biomolecular interaction analysis. To account for non-specific binding and bulk refractive index shifts, the final binding data were reported as a subtracted sensorgram (Relative RU = FC2 − FC1). The final response of bound streptavidin was 595 and 581 RU. To capture the biotinylated PolyPIPosome (20 µM, Echelon Biosciences, Salt Lake City, UT, USA), the chip was preconditioned with 1 M NaCl, 50 mM NaOH for 60 s at 30 µL/min. The biotinylated PolyPIPosome was then injected for 120 s at 10 µL/min to reach a final response of 979 RU. The binding analysis was carried out at 25 °C with HBS-N (10 mM HEPES, 150 mM NaCl, Cytiva cat# BR100670) as running buffer. Eight 3-fold dilutions of RTP004 (41.2 to 90,000 nM) were performed in HBS-N without BoNT/A1. Again, eight 3-fold dilutions of RTP004 were performed in HBS-N, and BoNT/A1 was added to reach a final concentration of 41.2 to 90,000 nM of RTP004 and 5 µM BoNT/A1 for every well. As a negative control, 5 µM of BoNT/A1 without RTP004 was analyzed. The samples were incubated at room temperature for 3 h. Each injection had a contact time of 120 s and dissociation time of 300 s at a flow rate of 30 µL/min. MgCl_2_ 4 M was used to regenerate the chip between injections and was injected at 30 µL/min for 30 s followed by a buffer wash and 300 s stabilization period. The binding levels of the BoNT/A1 + RTP004 samples were corrected for residual RTP004 binding by subtracting the binding levels of RTP004 alone from the binding levels of BoNT/A1 + RTP004.

### 5.5. SPR Binding Assay Analyzing Dissociation of RTP004 from BoNT/A1

BoNT/A1 (Revance Therapeutics) was directly immobilized onto Series S sensor chip C1 (Cytiva, cat# BR100535) using the same amine coupling as described above. BoNT/A1 was immobilized to a response signal of 348 RU. An EDC/NHS blank immobilization was used as a reference. To account for non-specific binding and bulk refractive index shifts, the final binding data were reported as a subtracted sensorgram (Relative RU = FC2 − FC1). RTP004 was diluted to 1000 nM in HBS-EP+ and injected for a contact time of 120 s at a flow rate of 30 µL/min followed by a dissociation time of 60 s. The various wash buffers tested were injected for 300 s at a flow rate of 30 µL/min. The relative RU was recorded after this wash step. At completion of each cycle, 4M MgCl_2_ was used as a regeneration buffer with a contact time of 30 s at a flow rate of 30 µL/min. The stability of the interaction was quantified by recording the relative response signal at two critical intervals: immediately following the primary binding event and initial wash and following the conclusion of the secondary 300 s wash step. The percentage of RTP004 dissociation was determined by calculating the ratio of the residual binding signal to the initial binding level.

### 5.6. Synaptosome Isolation and Binding Assay

The experiments on the effect of RTP004 on binding of BoNT/A1 H_C_ to rat synaptosomal cell membranes were conducted at toxogen GmbH. Synaptosomes were generated from rat brain homogenate as previously described [39,40]. Briefly, brains from Wistar rats were dissected and the cerebellum, pons, and medulla were discarded. The remaining brain was homogenized in homogenization buffer (320 mM sucrose, 5 mM HEPES-NaOH, pH 7.3) supplemented with protease inhibitors (Complete EDTA-free, Roche, Basel, Switzerland). Homogenate was centrifuged at 800× *g* for 10 min at 4 °C followed by centrifugation of the supernatant at 12,000× *g* for 15 min at 4 °C. The pellet was washed twice with homogenization buffer and afterwards the resuspended pellet was added to a discontinuous gradient of Percoll (10%, 15%, and 23%). The Percoll gradient was centrifuged for 7 min at 34,000× *g*. Synaptosomes were located between the Percoll interfaces (10% and 15%, and 15% and 23%). The synaptosomes were collected and placed into physiological buffer (PB; 132 mM NaCl, 4.8 mM KCl, 1.1 mM CaCl_2_, 20 mM HEPES, 2.4 mM MgSO_4_·7H_2_O, 2.4 mM glucose, pH 7.4). The optical density of the synaptosomes was measured at 660 nm and set to 0.3–0.4.

^35^S-labeled BoNT/A1 receptor-binding domain (H_C_) was synthesized in vitro from a pSP72 derivative using TnT^®^ Quick Coupled Transcription/Translation System (Promega, Walldorf, Germany) supplemented with L-[^35^S]-methionine. Synaptosomes (20 µL) were preincubated with or without 9 μg/mL RTP004 in binding buffer (PB + 4 mM histidine, 3.6 mM sucrose, 0.01% Tween-20, pH 6.85) for 15 min. After one washing step with binding buffer, 10 µL of ^35^S-labeled BoNT/A1 H_C_ was incubated with the synaptosomes (±9 μg/mL RTP004) in a final volume of 100 μL adjusted with binding buffer. Synaptosome binding assays were performed using a fixed aliquot of in vitro-translated ^35^S-labeled Hc, following previously established methodologies for characterizing ganglioside-dependent binding of Hcc domains of clostridial neurotoxins [39,40]. Binding was assessed as a function of incubation time and quantified relative to the L-[^35^S]-methionine. Because in vitro translation efficiency and the proportion of full-length product can vary, an exact molar concentration of Hc was not determined. Therefore, radioactive signal reflects incorporated label rather than absolute protein mass. A time-course experimental design was used to evaluate membrane association and accumulation of HcA under these conditions. Samples were incubated at 0 °C on ice and sampled at different time intervals. After incubation, the samples were resuspended twice, washed with PB buffer, centrifuged, and finally dissolved with PB supplemented with 0.01% Tween-20 pH 7.4 and 1.5-fold Laemmli buffer. Samples were incubated at 37 °C for 20 min and analyzed by 12.5% SDS-polyacrylamide gel electrophoresis, together with a 10 µL aliquot of ^35^S-labeled BoNT/A1 H_C_ as a reference. Bound ^35^S-labeled BoNT/A1 H_C_ was visualized using the Typhoon FLA-9000 laser scanner (GE HealthCare, Chicago, IL, USA) and quantified using Multi Gauge 3.2 software (Fujifilm, Tokyo, Japan).

### 5.7. Cell Culture

Human neuroblastoma SiMa cells were purchased from the German collection of Microorganism and Cell culture GmbH (DSMZ; Braunschweig, Germany) and were maintained in Roswell Park Memorial Institute (RPMI) 1640 Medium (Gibco, Dublin, Ireland) supplemented with 10% fetal bovine serum (FBS; Gibco, Waltham, MA, USA). Cells were passaged every 3–4 days and media changed every 2–3 days. All cells were maintained at 37 °C, 5% CO_2_ and were not used for more than 15 passages.

### 5.8. In Cell Western Assay

SiMa cells were plated in a 96-well plate, at 2 × 10^5^ cells/well, in growth medium (RPMI-1640 + 10% FBS) and allowed to attach for 2 days. On the treatment day, BoNT/A1 was labeled with CF^®^-790 Infrared dye Mix-n-Stain Antibody Labeling kit (Biotium) according to the manufacturer’s protocol. Cells were washed with serum-free medium (SFM) and treated with 1 μg/mL labeled BoNT/A1 and increasing concentrations of RTP004 or HSA prepared in SFM, and allowed to incubate at 37 °C and 5% CO_2_ for 1 h. After incubation with labeled toxin, cells were washed with 1X Dulbecco’s phosphate-buffered saline (DPBS) and fixed with 4% paraformaldehyde solution for 15 min at room temperature. Fixed cells were then incubated with 5 μM DRAQ5 fluorescent probe solution (Novus Biologicals, Centennial, CO, USA) for normalization using the 700 nm channel, and the plate was scanned dry on an Odyssey CLx Imager scanner (LI-COR^®^, Lincoln, NE, USA). Quantification was performed with Image Studio™ software (version 6.1) (LI-COR), and the BoNT/A1 signal (800 nm channel) was normalized to the DRAQ5 signal (700 nm channel).

### 5.9. SNAP-25 Cleavage Assay

SiMa cells were seeded in 6-well plates (Corning; Corning, NY, USA) at 1 × 10^6^ cells/well in 2 mL growth medium and incubated for 3 days. Cells were washed in SFM and treated with a fixed concentration of BoNT/A1 (100 ng/mL or 1 µg/mL) and increasing concentrations of RTP004 (1.5 to 25 µg/mL), scrRTP004 (15 µg/mL), or HSA (1.5 to 500 µg/mL) prepared in SFM, and incubated at 37 °C and 5% CO_2_ for 1 h. Cells were washed in SFM and returned to the same medium for 24 h. On the next day, cells were washed once with 1X DPBS and lysed in RIPA Extraction Reagent (Thermo Fisher Scientific) supplemented with 1X Halt Protease and Phosphatase Inhibitors Cocktail (Thermo Fisher Scientific). Protein concentration was measured using a bicinchoninic acid assay (Thermo Fisher Scientific) according to the manufacturer’s protocol. Lysates (40 μg total protein/lane) were resolved on a 4% to 20% Tris-glycine gel (Thermo Fisher Scientific) and run at 100 V for 2 h at room temperature followed by transfer to 0.22 μM nitrocellulose membranes (LI-COR). Membranes were blocked with Western blot blocking buffer (LI-COR) for 1 h at room temperature and incubated with primary antibodies mouse anti-SNAP-25 (Synaptic Systems, Göttingen, Germany; cat# 111 111, 1:2000), glyceraldehyde 3-phosphate dehydrogenase (GAPDH; Thermo Fisher Scientific, cat# PA1-16777, 1:500 or MA5-15738, 1:500) and actin-1 (ACTA1; Thermo Fisher Scientific, PA5-78715) overnight at 4 °C. Membranes were washed with 1X Tris Buffered Saline with Tween (TBS-T) (Thermo Fisher Scientific) and incubated in secondary antibody goat anti-mouse IRDye 800 or goat anti-rabbit IRDye (LI-COR) for 60 min. Membranes were washed in TBS-T and scanned in Odyssey Infrared Imaging system (LI-COR). Quantification of full-length and cleaved SNAP-25 signals was performed with Image Studio software. The amount of cleaved SNAP-25 was calculated as the ratio between cleaved and uncleaved bands.

### 5.10. Statistical Analyses

Differences between groups were evaluated using Student’s *t*-test. A *p*-value < 0.05) was considered statistically significant. For comparisons involving more than two groups, statistical significance was determined using one-way analysis of variance (ANOVA). A *p*-value < 0.05 was considered statistically significant.

## Data Availability

The original contributions presented in this study are included in the article. Further inquiries can be directed to the corresponding author.

## Supplementary Materials

The following supporting information can be downloaded at: https://www.mdpi.com/article/10.3390/toxins18030134/s1, Figure S1: SPR sensograms showing binding of HRP-labeled BoNT/A1 to RTP004-coated surfaces. SPR was used to assess the binding of BoNT/A1 to RTP004 immobilized at two surface densities. (A) HRP-labeled BoNT/A1 injected over RTP004 immobilized at 200 RU. (B) HRP-labeled BoNT/A1 injected over RTP004 immobilized at 1000 RU. Figure S2: SPR-Sensorgrams showing dissociation of RTP004 from BoNT/A under varying buffer conditions. BoNT/A1 was immobilized via EDC/NHS-mediated amine coupling to 348 RU. RTP004 was injected for a 120 s association phase, followed by a 60 s dissociation period in running buffer. Experimental wash buffers (acetate buffer at pH 5.5, 5.0 or 4.5, with 0–175 mM NaCl) were then applied for 300 s. Relative binding responses (FC2 − FC1) were recorded immediately after the primary binding event and initial wash (represented by the black arrow) and following the conclusion of the secondary 300 s wash step (represented by the red arrow). The stability of the interaction was quantified by recording the relative response signal at two critical intervals. The percentage of RTP004 dissociation was determined by calculating the ratio of the residual binding signal to the initial binding level.
