# Supplementary material for: RTP004 Peptide Binds to Botulinum Neurotoxin, Increases Cell Surface Binding, and Enhances Cellular SNAP-25 Cleavage
Source: Toxins (Basel). 2026 Mar 10;18(3):134. doi: 10.3390/toxins18030134 (PMC13030680; doi:10.3390/toxins18030134)
Supplement: Supplementary file 1 [file toxins-18-00134-s001.zip › DAXI MoA Fig S1_ProofUpdates.pdf]

(A)

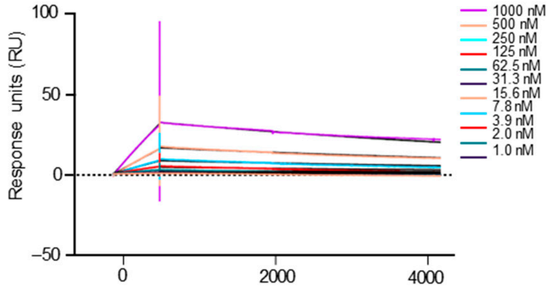

	K_D (M)	k_a(1/Ms)	k_d (1/s)	R_{max} (RU)	Chi² (RU²)
SPR Binding	1.381E-6	98.63	1.362E-4	32	0.815

(B)

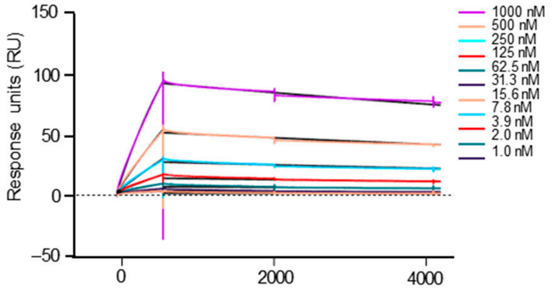

	K_D (M)	k_a(1/Ms)	k_d (1/s)	R_{max} (RU)	Chi² (RU²)
SPR Binding	7.319E-8	818.4	5.990E-5	88	5.19

Supplementary Figure S1: SPR sensograms showing binding of BoNT/A1 to RTP004-coated surfaces. SPR was used to assess the binding of BoNT/A1 to RTP004 immobilized at two surface densities. (A) HRP-labeled BoNT/A1 binding to RTP004 immobilized at 200 RU. (B) HRP-labeled BoNT/A1 binding to RTP004 immobilized at 1000 RU. Abbreviations: RU—response units; SPR—surface plasmon resonance.
