# Supplementary material for: RTP004 Peptide Binds to Botulinum Neurotoxin, Increases Cell Surface Binding, and Enhances Cellular SNAP-25 Cleavage
Source: Toxins (Basel). 2026 Mar 10;18(3):134. doi: 10.3390/toxins18030134 (PMC13030680; doi:10.3390/toxins18030134)
Supplement: Supplementary file 1 [file toxins-18-00134-s001.zip › DAXI MoA Fig S2_ProofUpdates.pdf]

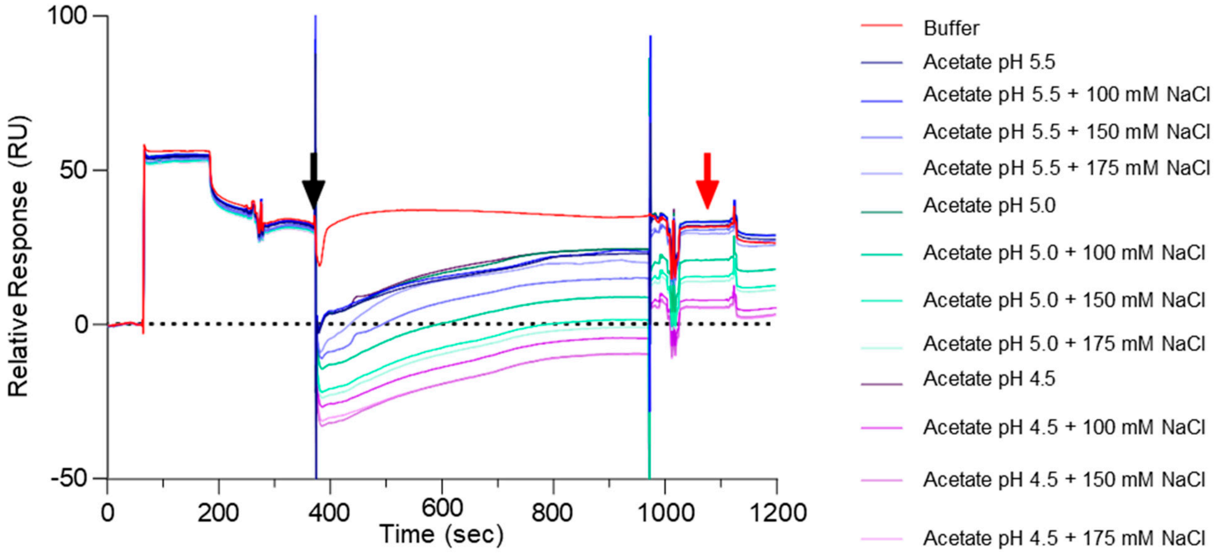

Supplementary Figure S2: SPR-Sensorgrams showing dissociation of RTP004 from BoNT/A under varying buffer conditions. BoNT/A1 was immobilized via EDC/NHS-mediated amine coupling to 348 RU. RTP004 was injected for a 120 s association phase, followed by a 60 s dissociation period in running buffer. Experimental wash buffers (acetate buffer at pH 5.5, 5.0 or 4.5, with 0-175 mM NaCl) were then applied for 300 s. Relative binding responses (FC2 – FC1) were recorded immediately after the primary binding event and initial wash (represented by the black arrow) and following the conclusion of the secondary 300 s wash step (represented by the red arrow). The stability of the interaction was quantified by recording the relative response signal at two critical intervals. The percentage of RTP004 dissociation was determined by calculating the ratio of the residual binding signal to the initial binding level. Abbreviations: NaCl—sodium chloride; RU—response units.
